# TGF‐β–responsive CAR‐T cells promote anti‐tumor immune function

**DOI:** 10.1002/btm2.10097

**Published:** 2018-07-27

**Authors:** Andrew J. Hou, ZeNan L. Chang, Michael H. Lorenzini, Eugenia Zah, Yvonne Y. Chen

**Affiliations:** ^1^ Dept. of Chemical and Biomolecular Engineering University of California, Los Angeles Los Angeles CA 90095; ^2^ Molecular Biology Institute University of California, Los Angeles Los Angeles CA 90095; ^3^ Dept. of Bioengineering University of California, Los Angeles Los Angeles CA 90095; ^4^ Parker Institute for Cancer Immunotherapy Center at UCLA Los Angeles CA 90095

**Keywords:** adoptive T‐cell therapy, chimeric antigen receptor, immunosuppression, immunotherapy, TGF‐β

## Abstract

A chimeric antigen receptor (CAR) that responds to transforming growth factor beta (TGF‐β) enables the engineering of T cells that convert this immunosuppressive cytokine into a potent T‐cell stimulant. However, clinical translation of TGF‐β CAR‐T cells for cancer therapy requires the ability to productively combine TGF‐β responsiveness with tumor‐targeting specificity. Furthermore, the potential concern that contaminating, TGF‐β?producing regulatory T (Treg) cells may preferentially expand during TGF‐β CAR‐T cell manufacturing and suppress effector T (Teff) cells demands careful evaluation. Here, we demonstrate that TGF‐β CAR‐T cells significantly improve the anti‐tumor efficacy of neighboring cytotoxic T cells. Furthermore, the introduction of TGF‐β CARs into mixed T‐cell populations does not result in the preferential expansion of Treg cells, nor do TGF‐β CAR‐Treg cells cause CAR‐mediated suppression of Teff cells. These results support the utility of incorporating TGF‐β CARs in the development of adoptive T‐cell therapy for cancer.

## 1 INTRODUCTION

Adoptive T‐cell immunotherapy has produced promising results in the treatment of advanced B‐cell malignancies, leading to the approval of anti‐CD19 chimeric antigen receptor (CAR)‐T cell therapies by the United States Food and Drug Administration in 2017.^1^, ^2^ However, successful treatment of solid tumors has been more elusive, in part due to the highly immunosuppressive nature of tumor microenvironments.^3^ One prominent mechanism by which tumor cells evade immune surveillance is the secretion of transforming growth factor beta (TGF‐β).^4^ TGF‐β has been shown to not only directly suppress T‐cell effector function^5^, ^6^ but also drive T‐cell differentiation into the regulatory phenotype.^7^ Regulatory T (Treg) cells are, in turn, able to produce TGF‐β and further promote tumor tolerance.^8^, ^9^

Because of its suppressive role in the tumor microenvironment, TGF‐β has been targeted in several studies seeking to boost anti‐tumor immunity. For example, monoclonal antibodies targeting TGF‐β and TGF‐β receptor chain 2 (TGFBR2) have been extensively studied in preclinical and early‐phase clinical studies.^10^ Other studies have more specifically targeted TGF‐β signaling within the tumor microenvironment with a TGF‐β dominant‐negative receptor (TGF‐β DNR), which renders transduced tumor‐specific T cells unresponsive to TGF‐β.^11^, ^12^, ^13^ We recently described a novel CAR that responds to TGF‐β (TGF‐β CAR), demonstrating the ability to not only inhibit endogenous TGF‐β signaling in T cells but also convert TGF‐β into a potent T‐cell stimulant.^14^ Indeed, TGF‐β CAR‐T cells proliferate robustly and secrete Th1 cytokines in the presence of TGF‐β.

Here, we demonstrate that TGF‐β CAR‐T cells can also protect neighboring immune cells from the suppressive effects of TGF‐β by enabling tumor‐targeted CD8^+^ T cells to retain cytolytic activity in the presence of TGF‐β, and by discouraging CD4^+^ T cells from TGF‐β–induced Treg differentiation. Furthermore, we show that “contaminating” Tregs within bulk T cells transduced with the TGF‐β CAR are not a liability, as TGF‐β CAR‐transduced Tregs do not preferentially expand or exert detrimental immune suppression. Taken together, our results suggest that the TGF‐β CAR can safely and effectively boost the anti‐tumor efficacy of T‐cell therapy.

## 2 MATERIALS AND METHODS

### 2.1 DNA constructs

TGF‐β–specific CARs with short and long spacers (IgG4 hinge and IgG4 hinge‐CH2‐CH3, respectively), as well as the scFv‐less CAR, were constructed as previously described.^14^ The CARs contain the CD28 transmembrane domain, the CD28 cytosolic tail with GG mutations to enhance CAR surface expression,^15^ and the CD3ζ cytosolic domain. The NY‐ESO‐1 TCR was a gift from Dr. Antoni Ribas (University of California, Los Angeles).^16^ The CD19‐ and CD20‐binding CARs contain the 4‐1BB and CD3ζ intracellular domains as previously described.^17^ The TGF‐β DNR encodes the first 199 amino acids of TGFBR2. All receptors were linked by the “self‐cleaving” T2A sequence to a truncated epidermal growth factor receptor (EGFRt), a non‐signaling transduction marker that also facilitates sorting of CAR‐expressing cells.^18^

### 2.2 Cell lines

Raji cells and TM‐LCLs, an Epstein‐Barr virus‐transformed lymphoblastoid cell line, were gifts from Dr. Michael Jensen (Seattle Children's Research Institute). M407 human melanoma cells stably transfected with a nuclear‐localizing red fluorescent protein (RFP) were a gift from Dr. Antoni Ribas. EGFP NFAT reporter Jurkat cells were a gift from Dr. Arthur Weiss (University of California, San Francisco). Cell lines were maintained in complete RPMI (RPMI1640 (Lonza) + 10% heat‐inactivated FBS (Gibco)).

### 2.3 Generation of CAR‐expressing primary human T cells

Lentivirus was produced as previously described.^17^ CD4^+^ or CD8^+^ T cells were isolated with the RosetteSep CD4^+^ or CD8^+^ Human T‐Cell Enrichment Cocktail (STEMCELL Technologies) from healthy donor whole blood obtained from the UCLA Blood and Platelet Center. T cells were then stimulated with CD3/CD28 Dynabeads (Thermo Fisher Scientific) at a 1:1 cell:bead ratio for 2 days and transduced with lentivirus at a multiplicity of infection (MOI) of 1.5–3. T cells were cultured in complete RPMI and fed 50 U/mL IL‐2 (Thermo Fisher Scientific) and 1 ng/mL IL‐15 (Miltenyi Biotec) every 2–3 days. Dynabeads were removed after 9 days of culture. Transduced cells were enriched by magnetic bead‐based sorting (Miltenyi Biotec) and kept in culture with IL‐2/IL‐15 supplementation every 2–3 days.

For experiments involving Treg cells, CD4^+^ T cells isolated as mentioned above were stained with fluorescently conjugated anti‐CD4 (clone RPA‐T4, BioLegend), anti‐CD25 (clone BC96, BioLegend), and anti‐CD127 (clone A019D5, BioLegend) antibodies, and then Treg (CD4^+^CD25^hi^CD127^–^) and non‐Treg (CD4^+^CD25^–^) fractions were enriched on a BD FACSAria II cell sorter. Treg cells were stimulated with CD3/CD28 Dynabeads at a 1:1 cell:bead ratio for 2 days and then transduced with lentivirus at an MOI of 5. Tregs were cultured in complete RPMI supplemented with 100 nM rapamycin and fed 300 U/mL IL‐2 every 2–3 days. Dynabeads were removed after 10 days of culture, and cells were subsequently re‐stimulated with Dynabeads at a 2:1 cell:bead ratio. Cells were then cultured in complete RPMI without rapamycin, and IL‐2 was supplemented every 2–3 days. In some experiments, cultures were also supplemented with 5 ng/mL TGF‐β after re‐stimulation. Dynabeads were removed on day 20 or 21 of culture. Following Dynabead removal, Tregs were rested in IL‐2–free medium for 24 hr prior to use in downstream experiments.

A note on TGF‐β concentration used in this study: To our knowledge, there has been no report of typical active TGF‐β concentrations found in *tumor tissue*. Instead, published studies have relied on ELISA or receptor binding assays performed on *blood plasma*, with widely varying results (0.5–25 ng/mL in human plasma).^19^ It has been shown that TGF‐β concentrations are significantly higher in plasma samples from cancer patients compared to healthy controls,^20^ but the precise level of active TGF‐β at tumor local environments remains unknown. Active TGF‐β concentrations are expected to be higher at tumor sites than in plasma, as solid tumors are known to secrete TGF‐β, and latent forms of TGF‐β (produced by either tumor or other cell types) are known to be activated by tumor‐associated metalloproteases.^21^ Based on available information, we have chosen to use 5 ng/mL as the standard TGF‐β input concentration because (a) it is within the reported range of physiological TGF‐β concentrations, (b) it is consistent with the conditions used by various research groups studying TGF‐β signaling in T cells,^12^, ^22^, ^23^, ^24^ and (c) this input level has been verified to induce endogenous TGF‐β signaling and functional defects in primary human T cells.^14^

### 2.4 T‐cell cytotoxicity assays

An automated live‐cell imaging system (IncuCyte, Essen BioScience) was used to evaluate T‐cell cytotoxicity dynamics against the adherent NY‐ESO‐1^+^ RFP^+^ M407 cell line, following a previously described protocol.^25^ RFP^+^ M407 cells were seeded overnight at 10^4^ cells/100 μL/well in flat‐bottom 96‐well plates. The next day, T‐cell mixtures (2.5 × 10^4^ CD8^+^ T cells expressing the NY‐ESO‐1 TCR plus 2.5 × 10^4^ CD4^+^ T cells expressing the indicated constructs) were applied in 100 μL volumes to target cells in triplicate, with or without 5 ng/mL TGF‐β. In order to observe a clear TGF‐β–induced defect in T‐cell cytotoxicity, T cells were collected 48 hr after the first challenge and applied to a new batch of tumor cells (seeded the night before) for a second challenge, with or without 5 ng/mL TGF‐β. The amount of red fluorescence, measured every 2 hr and normalized to the time 0 fluorescence, indicated the proportion of live tumor cells remaining. Log‐linear models were applied to cytotoxicity dynamics data using R 3.3.2 software.

An electrical impedance‐based tumor cell culture system (xCELLigence, ACEA Biosciences) was used to evaluate T‐cell cytotoxicity dynamics against the CD20^+^ Raji cell line. Though normally suspension cells, Raji cells were immobilized onto 96‐well, electrode‐bottomed plates pre‐coated with anti‐CD40 antibodies at 3 × 10^4^ cells/100 μL/well. The next day, T‐cell mixtures (3 × 10^4^ CD8^+^ T cells expressing the CD20 CAR plus 3 × 10^4^ CD4^+^ T cells expressing the indicated constructs) were added in quadruplicate, with or without 5 ng/mL TGF‐β. Forty‐eight hours later, T cells were collected and applied to a new batch of tumor cells (seeded the night before) for the second challenge, with or without 5 ng/mL TGF‐β. The cell index, a measure of electrode impedance correlating to Raji‐cell viability, was measured every 15 min, normalized to the time 0 cell index, and used as an indicator for the proportion of live tumor cells remaining. Log‐linear models were applied to cytotoxicity dynamics data using R 3.3.2.

### 2.5 Treg cell induction assay

Previously unstimulated CD4^+^ T cells were seeded in OKT3‐coated 96‐well U‐bottom plates at 5 × 10^4^ cells/100 μL/well with 20 U/mL IL‐2, 1 μg/mL CD28 agonist antibody (clone CD28.2, BioLegend), and 0 or 5 ng/mL TGF‐β. After 1 day, the samples were supplemented with donor‐matched CD4^+^ T cells expressing the indicated constructs. The supplementary cells were stained with 0.5 μM CellTrace Violet (CTV) and added at 5 × 10^4^ cells/50 μL/well in media supplemented with 20 U/mL IL‐2, 1 μg/mL CD28 agonist antibody, and 0 or 5 ng/mL TGF‐β. On day 5 of the assay, cells were stained with anti‐CD25 antibody, fixed and permeabilized (True‐Nuclear Transcription Factor Buffer Set, BioLegend), and stained with anti‐FOXP3 antibody (clone 206D, BioLegend).

### 2.6 NFAT reporter assays

NFAT EGFP reporter Jurkat cells transduced with the TGF‐β CAR were seeded at 5–10 × 10^4^ cells/100 μL/well in triplicate in 96‐well plates, with indicated levels of human TGF‐β or human latent latency associated peptide (LAP)‐bound TGF‐β (BioLegend). Reporter induction was assessed by flow cytometry after 17 hr at 37°C.

### 2.7 Treg suppression assays

In experiments testing TCR‐mediated suppression, CD4^+^CD25^−^ Teffs that were never activated *in vitro* were stained with 1.25 μM CFSE. CFSE‐labeled Teffs were seeded in OKT3‐coated 96‐well U‐bottom plates at 5 × 10^4^ cells/well with 1 μg/mL CD28 agonist antibody (clone CD28.2, BioLegend), and 0 or 5 ng/mL TGF‐β. Tregs were added to each well at a 1:1 Treg:Teff ratio.

In experiments testing CAR‐mediated suppression, CD19 CAR‐transduced CD4^+^CD25^–^ Teffs were stained with 1.25 μM CFSE, and Tregs were stained with 1.25 μM CTV. CFSE‐labeled Teffs were seeded in 96‐well U‐bottom plates at 5 × 10^4^ cells/well with 1 × 10^5^ irradiated TM‐LCL cells and 0, 5, or 10 ng/mL TGF‐β. CTV‐labeled Tregs were added to each well at a 1:1 Treg:Teff ratio.

### 2.8 Statistical analyses

**Figure 1:**
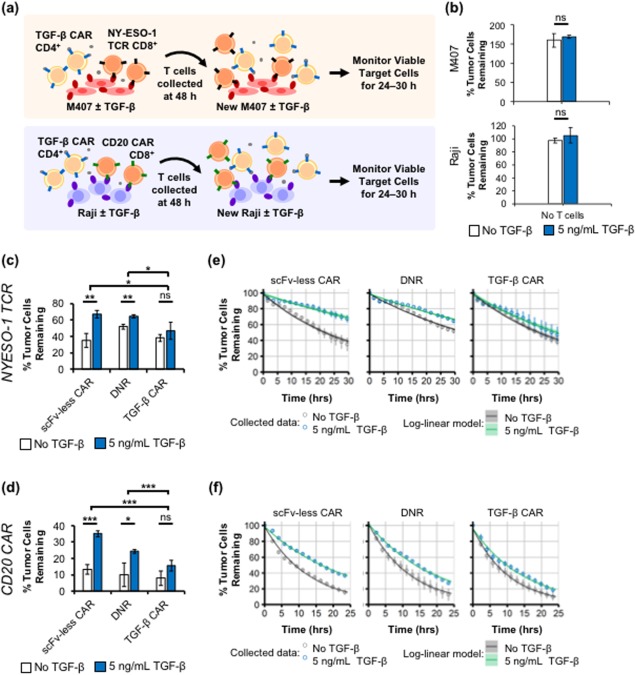
TGF‐β CAR‐T cells reduce TGF‐β–mediated suppression of CD8^+^ T‐cell cytotoxicity. (a) Schematic of assay setup. CD4^+^ T cells expressing an scFv‐less CAR, TGF‐β DNR, or TGF‐β CAR were co‐cultured with donor‐matched CD8^+^ T cells expressing an NY‐ESO‐1 TCR or CD20 CAR, and challenged twice with cognate tumor cells (NY‐ESO‐1^+^ M407 or CD20^+^ Raji, respectively) in the presence or absence of TGF‐β. (b) In the absence of T cells, TGF‐β does not impact the expansion of NY‐ESO‐1^+^ M407 and CD20^+^ Raji cells within the time‐scale of the assay. The % of tumor cells remaining relative to the number of tumor cells at time 0 is shown. NY‐ESO‐1+ M407 melanoma cells were cultured for 29.5 hr and CD20^+^ Raji cells were cultured for 24 hr. (c,d) Percent of tumor cells remaining quantified at the end of the second challenge. (e,f) Time‐courses of the % tumor cells remaining during the second challenge, overlaid with log‐linear fits of tumor‐cell killing dynamics. Shading around the line indicates the 99% confidence band of the fit. Model parameters are presented in Table 1. For visibility, every seventh time point is shown in (f). Averages of (c,e) triplicates or (d,f) quadruplicates are shown with error bars representing ± 1 standard deviation (SD). Statistics for TGF‐β–dependent changes are calculated by two‐tailed Student's *t* tests with the Sidak correction for multiple comparisons. ANOVAs of TGF‐β–exposed cell mixtures yielded significant variation among the different mixtures with (c) F = 6.8, df = 3, *p* < .05; and (d) F = 43.6, df = 3, *p* < .001. * *p* < .05, ** *p* < .01, and *** *p* < .001

**Figure 2:**
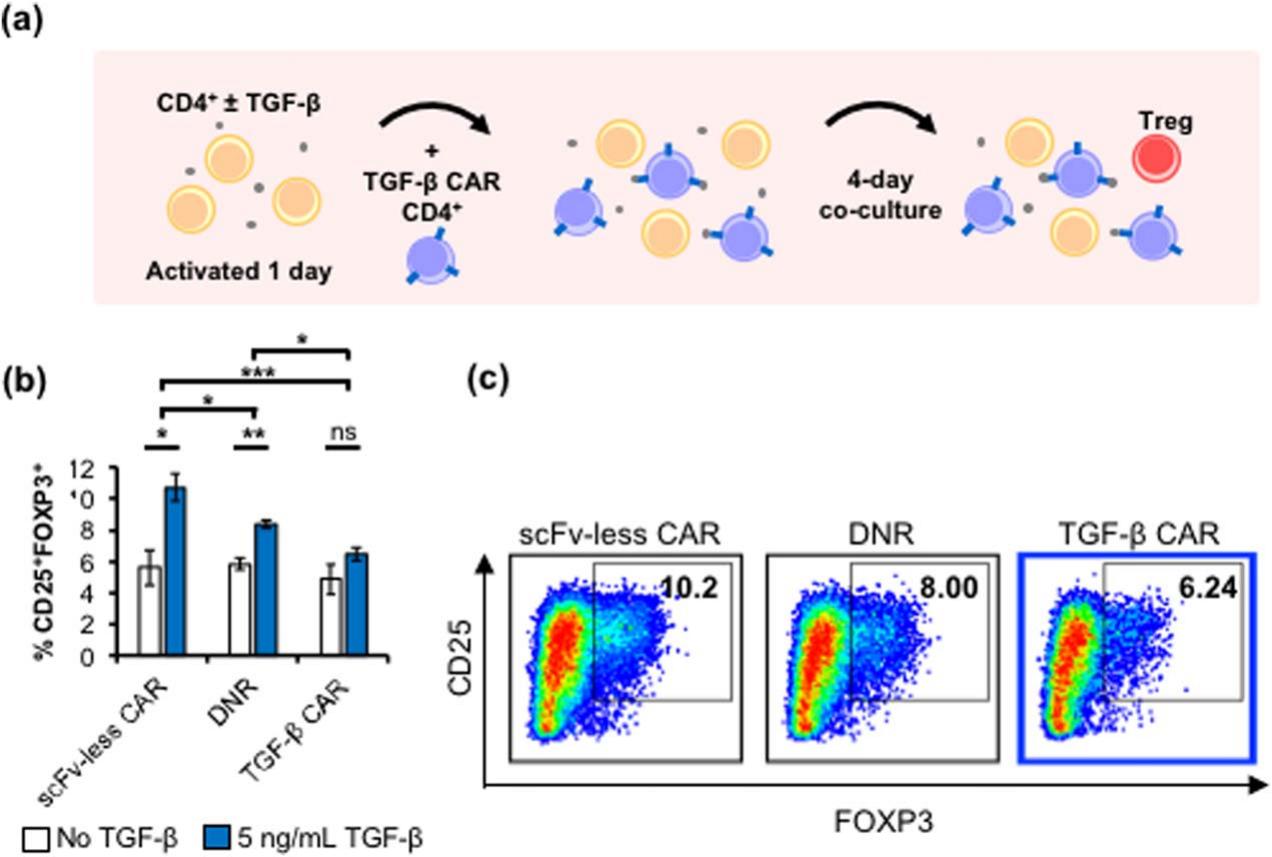
TGF‐β CAR‐T cells reduce the induction of Treg differentiation. (a) Schematic of assay setup. Previously unactivated CD4^+^ T cells were cultured in OKT3‐coated wells with IL‐2 and anti‐CD28, with or without TGF‐β for 24 hr. The wells were subsequently supplemented with donor‐matched, CTV‐stained CD4^+^ T cells expressing the scFv‐less CAR, TGF‐β DNR, or TGF‐β CAR. The emergence of Treg cells was quantified 4 days later. (b) Induction of CD25^+^FOXP3^+^ subpopulations in CD4^+^ T cells in cultures with or without TGF‐β, after the addition of CD4^+^ T cells expressing the different synthetic receptors. Averages of triplicates are shown with error bars representing ± 1 SD. Statistics for TGF‐β–dependent changes are calculated by two‐tailed Student's *t* test with the Sidak correction for multiple comparisons. ANOVAs of TGF‐β–exposed cell mixtures yielded significant variation among the different mixtures with F = 22.7, df = 3, *p* < .001. Pairwise contrasts are evaluated post‐hoc by Tukey's test. * *p* < .05, ** *p* < .01, and *** *p* < .001. (c) Representative CD25 versus FOXP3 scatters are shown for the viable/singlet/CTV^–^ gated population

Statistical tests were performed in Excel and R 3.3.2. Student's *t* tests with unequal variances were used to compare continuous variables between two groups, with the Sidak correction for multiple comparisons. Analyses of variance (ANOVAs) were used to assess variation among more than two groups, with post‐hoc pairwise comparisons by Dunnett's test when contrasting multiple factors with a single standard factor (Figure 1c,d) or by Tukey's test when contrasting multiple factors with more than one standard factor (Figure 2b). All tests were two‐tailed with a hypothesis‐specific family alpha level of 0.05.

## 3 RESULTS

### 3.1 TGF‐β CAR‐T cells protect tumor‐targeting T cells from TGF‐β–mediated immunosuppression

An important advantage of the TGF‐β CAR is its ability to trigger effector functions that interface with other immune cells, a feature not available to the TGF‐β DNR. To characterize this property, a series of co‐culture assays were performed to investigate whether TGF‐β CAR‐T cells can indeed support the anti‐tumor immunity of nearby T cells. First, we found that CD4^+^ TGF‐β CAR‐T cells significantly reduced the ability of TGF‐β to impair the cytotoxicity of tumor‐targeting CD8^+^ T cells. CD8^+^ T cells engineered to express either an NY‐ESO‐1 TCR or a CD20 CAR were co‐incubated with donor‐matched CD4^+^ T cells expressing one of three constructs: (a) the TGF‐β CAR; (b) an scFv‐less CAR, which is identical to the TGF‐β CAR except it lacks the scFv domain and thus cannot bind TGF‐β; or (c) the TGF‐β DNR. Each T‐cell mixture was challenged with two rounds of cognate tumor cells (i.e., NY‐ESO‐1^+^ M407 melanoma or CD20^+^ Raji, neither of which shows changes in growth rate in response to TGF‐β alone) (Figure 1a,b). In this co‐culture, only the CD8^+^ T cells have tumor‐recognition capability, since the CD4^+^ T cells do not express a tumor‐antigen–specific receptor. Unique among the four T‐cell mixtures tested, samples containing TGF‐β CAR‐T cells showed no significant change in tumor‐killing capability despite the presence of TGF‐β, resulting in significantly lower tumor‐cell survival compared to samples expressing the scFv‐less CAR or DNR (Figure 1c–f). Cell‐killing kinetics were quantified by fitting the time‐course data to log‐linear models, which proportionally relate the rate of killing to the number of tumor cells remaining (Figure 1e,f). The results indicated that for both NY‐ESO‐1 TCR‐ and CD20 CAR‐expressing cytotoxic T cells, pairing with CD4^+^ TGF‐β CAR‐T cells resulted in the least amount of TGF‐β–induced loss of cytotoxicity and the highest killing rate in the presence of TGF‐β (Table 1).

**Table 1:** Rate constants for tumor‐cell killing dynamics

		Kill rate constants ± SE (×10^−2^/hr)		
CD8^+^ cell line	CD4^+^ cell line	No TGF‐β	5 ng/mL TGF‐β	Difference
NY‐ESO‐1 TCR	scFv‐less CAR	3.13 ± 0.09	1.21 ± 0.13	1.92 ± 0.09
	TGF‐β DNR	2.03 ± 0.05	1.33 ± 0.06	0.71 ± 0.04
	TGF‐β CAR	2.96 ± 0.11	2.30 ± 0.15	0.65 ± 0.11
CD20 CAR	scFv‐less CAR	7.66 ± 0.04	4.10 ± 0.06	3.56 ± 0.04
	TGF‐β DNR	8.26 ± 0.08	5.18 ± 0.11	3.08 ± 0.7
	TGF‐β CAR	9.41 ± 0.10	6.68 ± 0.14	2.73 ± 0.09

Log‐linear models, which state that the rate of killing is proportional to the number of remaining tumor cells, were applied to tumor‐cell death curves (Figure 1e,f) to estimate the TGF‐β–mediated difference in cytotoxicity rate constants of various T‐cell mixtures. All model parameters were estimated with high statistical confidence (*p* < 10^−7^). SE, standard error.

### 3.2 TGF‐β CAR‐T cells suppress TGF‐β–induced differentiation into Treg phenotype

We next evaluated the impact of TGF‐β CAR‐T cells on the differentiation of naïve T cells into the Treg phenotype, which suppresses tumor rejection.^3^ TGF‐β has been shown to promote Treg differentiation,^7^ and this behavior was confirmed through the following co‐culture experiment: previously unactivated CD4^+^ T cells were primed with IL‐2, anti‐CD28, and immobilized OKT3, with or without TGF‐β. One day later, donor‐matched, CTV dye‐labeled T cells expressing the scFv‐less CAR, TGF‐β DNR, or TGF‐β CAR were added (Figure 2a). After 4 days of co‐culture, a significant increase in the proportion of dye‐negative CD25^+^FOXP3^+^ cells was observed specifically in the presence of TGF‐β, and this Treg differentiation could not be prevented by the addition of T cells expressing the scFv‐less CAR or the DNR (Figure 2b). In contrast, T cells expressing TGF‐β CAR blocked Treg differentiation of nearby (CTV^−^) T cells, resulting in no significant increase in the proportion of Treg cells even in the presence of TGF‐β (Figure 2b,c). Altogether, the TGF‐β CAR‐T cells are superior to TGF‐β DNR‐expressing T cells in restricting the ability of TGF‐β to orchestrate immunosuppressive functions.

### 3.3 TGF‐β CAR does not respond to latent form of TGF‐β

**Figure 3:**
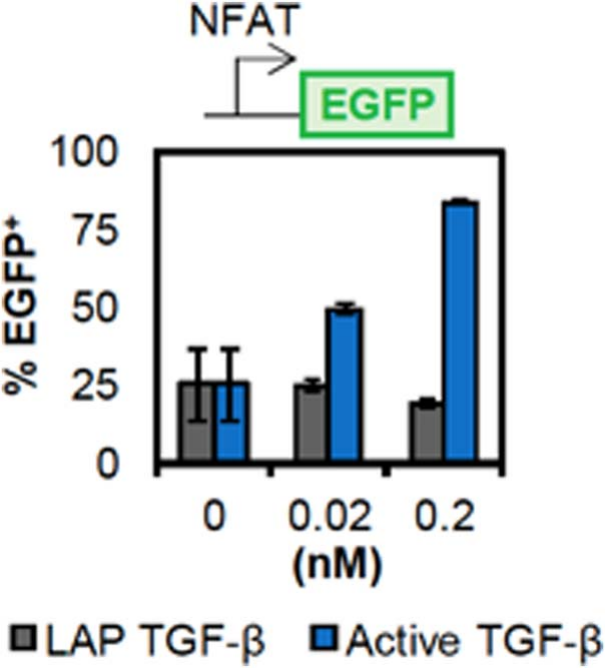
TGF‐β CAR‐T cells do not respond to the latent form of TGF‐β. Soluble TGF‐β in the active or latent (LAP‐bound) form was added at the specified concentrations to a Jurkat T‐cell line that stably expresses an EGFP reporter driven by an NFAT‐responsive promoter. EGFP expression was quantified by flow cytometry after 17 hr. The TGF‐β CAR‐T cells are only activated by the active form of TGF‐β

A potential concern around the clinical translation of TGF‐β CAR‐T cells is whether they would be non‐specifically activated in normal tissue, resulting in systemic toxicity. All three TGF‐β isoforms—TGF‐β1, TGF‐β2, and TGF‐β3—are produced as precursor molecules and are generally found in the latent form bound to a LAP.^26^ Activation of the TGF‐β homodimer requires the presence of metalloproteases or mechanical forces to induce the dissociation of the LAP. Therefore, only the latent and not the active form of TGF‐β is found at high levels in blood serum.^26^ We have confirmed that the TGF‐β CAR does not respond to the latent form of TGF‐β (Figure 3), thus reducing the risk of non‐specific, systemic activation of TGF‐β CAR‐T cells in non‐tumor tissue. Coupled with our previously reported observation that TGF‐β CAR activation is dependent on TGF‐β dose input,^14^ these results strongly support that TGF‐β CAR‐T cells would only be activated in areas of high TGF‐β local concentration, such as solid tumor microenvironments.^14^

### 3.4 TGF‐β CAR expression in Treg cells does not trigger CAR‐mediated suppression

At present, CAR‐T cell therapy is a personalized medical treatment, with unique cell products produced for each patient.^27^ Although a variety of cell‐manufacturing starting materials are used, including peripheral blood mononuclear cells (PBMCs), CD8‐sorted cells, or T cells sorted for either naive or memory phenotypes,^28^, ^29^, ^30^, ^31^ most manufacturing processes do not specifically deplete Treg cells. Since activated Treg cells secrete TGF‐β,^8^ and TGF‐β CAR signaling promotes robust T‐cell expansion,^14^ concerns arise of whether TGF‐β CAR expression would inadvertently enrich Treg cells in a T‐cell product, and whether TGF‐β CAR‐Treg cells would cause counterproductive CAR‐mediated immunosuppression.

**Figure 4:**
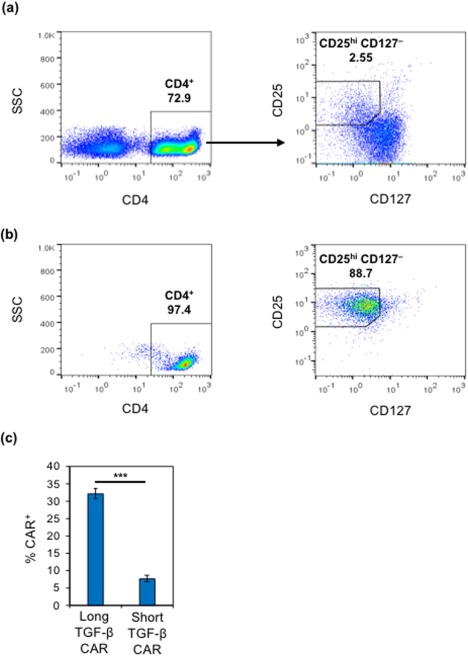
The TGF‐β CAR with a long spacer exhibits more efficient surface presentation in Tregs than the TGF‐β CAR with a short spacer. (a) Representative flow plots depicting gating strategy for the sorting of CD4^+^/CD25^hi^/CD127^–^ Tregs. (b) Representative flow plots depicting surface marker expression of cells immediately after sorting. (c) TGF‐β CAR‐transduced CD4^+^/CD25^hi^/CD127^–^‐sorted cells were stained for surface expression of FLAG‐tagged CARs. Averages of triplicates are shown, with error bars representing ± 1 SD. Statistics are calculated by a two‐tailed Student's *t* test. *** *p* < .001

To evaluate the effect of TGF‐β CAR expression specifically on Tregs, experiments were performed using FACS‐sorted CD4^+^/CD25^hi^/CD127^–^ cells (Figure 4a,b), which have been shown to be enriched in the Treg phenotype.^32^ Interestingly, unlike non‐Tregs,^14^ Treg cells consistently showed more efficient surface presentation of the TGF‐β CAR containing a long (229‐amino acid) extracellular spacer compared to an otherwise identical CAR containing a short (12‐amino acid) spacer (Figure 4c). Therefore, all subsequent experiments in Treg cells were performed with the long TGF‐β CAR.

**Figure 5:**
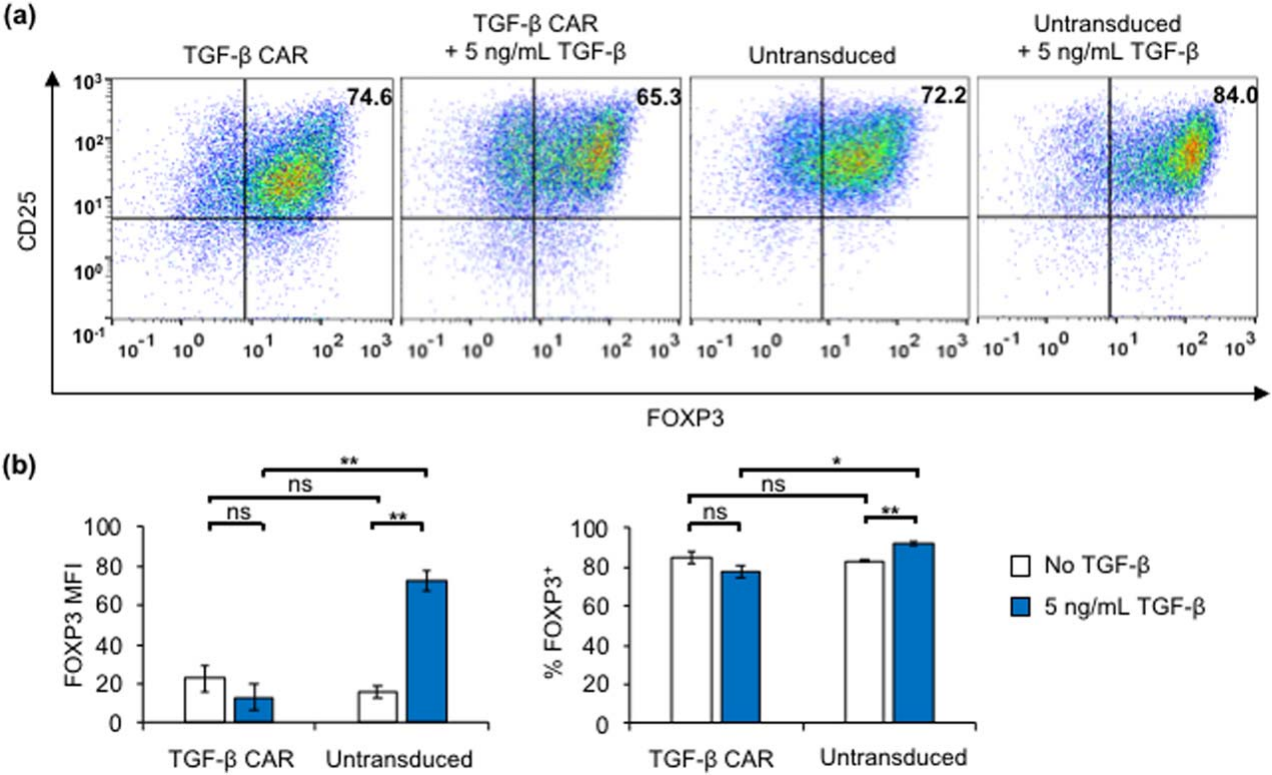
TGF‐β CAR expression and stimulation does not result in preferential expansion of FOXP3^+^ Tregs among CD4^+^/CD25^hi^/CD127^‐^‐sorted cells. (a) TGF‐β CAR‐transduced or untransduced CD4^+^/CD25^hi^/CD127^‐^‐sorted cells were expanded with Dynabeads only or Dynabeads plus 5 ng/mL TGF‐β for 20 days. Shown are representative scatterplots of FOXP3 versus CD25 expression corresponding to each transduction and expansion condition. (b) FOXP3 mean fluorescent intensity (MFI) and frequency of FOXP3^+^ cells corresponding to each transduction and expansion condition. Averages of triplicates are shown in (b), with error bars representing ± 1 SD. Statistics are calculated by two‐tailed Student's *t* test with the Sidak correction for multiple comparisons. * *p* < .05 and ** *p* < .01

The most definitive marker of Treg phenotype—FOXP3—is an intracellular protein that cannot be used for live‐cell sorting based on surface antibody staining. As a result, the expanded CD4^+^/CD25^hi^/CD127^–^‐sorted cell population included a residual fraction of FOXP3^−^ cells, regardless of whether the cells had been transduced with the TGF‐β CAR (Figure 5a). Consistent with previous observations (Figure 2b), the addition of TGF‐β resulted in an increase in FOXP3 expression in untransduced, CD4^+^/CD25^hi^/CD127^−^‐sorted cells, confirming that TGF‐β promotes the differentiation of non‐Treg cells into the Treg phenotype (Figure 5a,b). In contrast, TGF‐β–mediated induction of FOXP3 expression was not observed in CD4^+^/CD25^hi^/CD127^−^‐sorted cells transduced with the TGF‐β CAR. These results indicate that TGF‐β CAR expression does not promote preferential expansion of FOXP3^+^ Tregs over non‐Tregs. Instead, CAR expression appears to suppress the overall frequency of FOXP3^+^ Tregs in mixed T‐cell populations treated with TGF‐β (Figure 5b), likely by preventing the differentiation of non‐Treg cells into the regulatory phenotype as previously observed in Figure 2. We expect the suppression of FOXP3 upregulation to be specific to TGF‐β CARs as opposed to CARs in general, since CD19 CAR and scFv‐less CAR‐T cells are neither activated by TGF‐β, nor do they exhibit changes in endogenous TGF‐β signaling compared to mock‐transduced T cells.^14^

**Figure 6:**
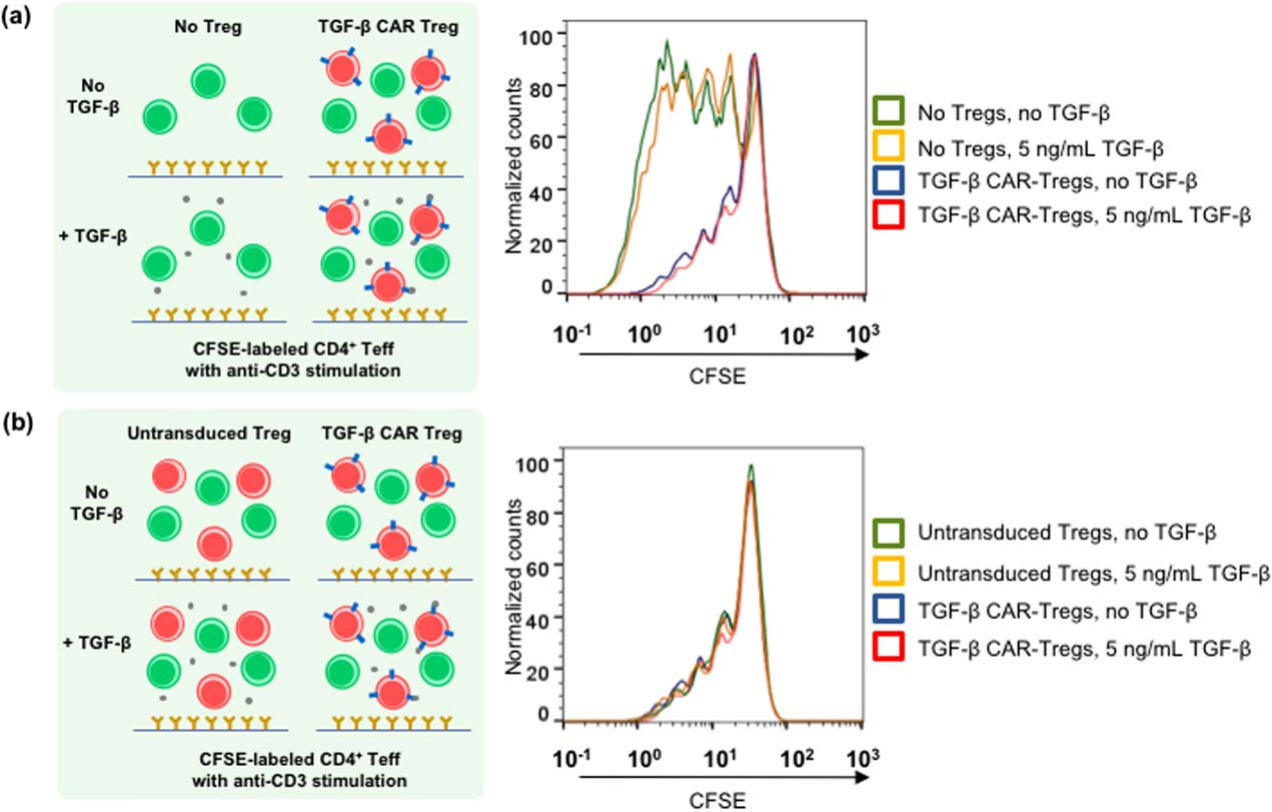
TGF‐β CAR‐transduced CD4^+^/CD25^hi^/CD127^–^‐sorted cells are suppressive when stimulated through the TCR. (a) CFSE‐labeled CD4^+^ Teffs that were not previously activated *in vitro* were cultured in OKT3‐coated wells with CD28 agonist antibody and either 0 or 5 ng/mL TGF‐β, with or without the addition of TGF‐β CAR‐transduced CD4^+^/CD25^hi^/CD127^–^‐sorted cells (referred to as TGF‐β CAR‐Tregs) at 1:1 Treg:Teff ratio. Representative histogram overlays of CFSE dilution are shown. (b) Co‐cultures were set up as described in (a), except all wells received Tregs that were either untransduced or transduced with the TGF‐β CAR. Representative histogram overlays of CFSE dilution are shown

We next evaluated whether Treg cells activated through TGF‐β CAR signaling would exert suppressive effects on nearby Teff cells. Co‐cultures were set up with CFSE‐labeled CD4^+^CD25^−^ Teff cells, anti‐CD3, and anti‐CD28, with or without TGF‐β CAR‐transduced CD4^+^/CD25^hi^/CD127^–^‐sorted cells (Figure 6a). In such co‐cultures, anti‐CD3 and anti‐CD28 served to activate both Treg and Teff cells by triggering TCR signaling. CFSE dilution in Teff cells was quantified after 96 hr of co‐culture, with results indicating clear suppression of Teff proliferation in the presence of CD4^+^/CD25^hi^/CD127^−^‐sorted cells (Figure 6a), confirming this population contained sufficient numbers of Treg cells to effectively suppress Teff proliferation upon TCR‐mediated T‐cell activation. Notably, addition of TGF‐β did not enhance suppression mediated by TGF‐β CAR‐transduced Treg cells (Figure 6a). Furthermore, TGF‐β CAR‐transduced Tregs were no more suppressive than untransduced Tregs (Figure 6b). These results indicate that the TGF‐β CAR itself does not boost the suppressive capacity of Treg cells.

**Figure 7:**
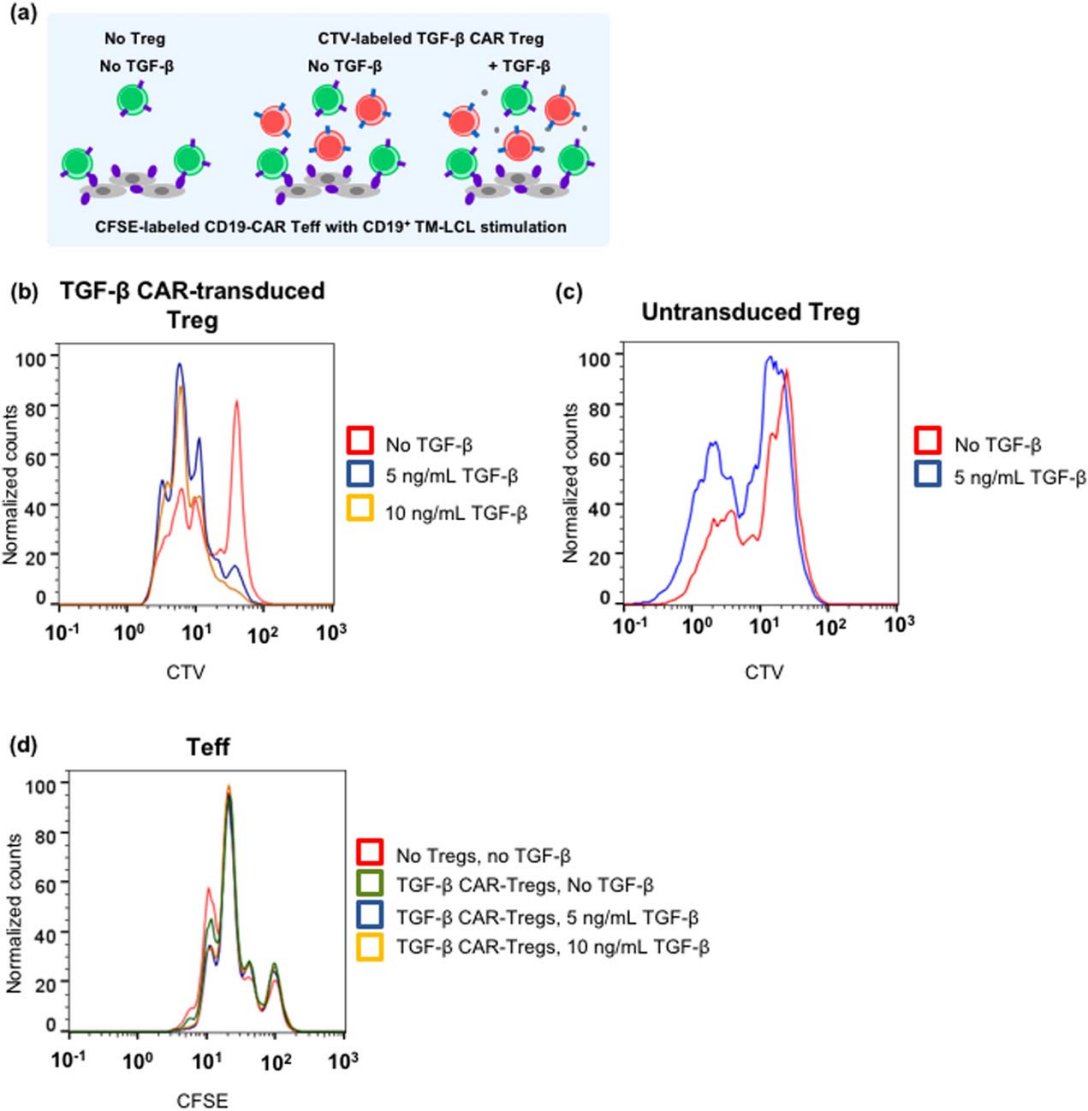
TGF‐β CAR‐transduced CD4^+^/CD25^hi^/CD127^‐^‐sorted cells (referred to as TGF‐β CAR‐Tregs) are not suppressive when stimulated through the CAR. (a) CFSE‐labeled CD19 CAR‐Teffs were co‐cultured with irradiated CD19^+^ TM‐LCLs, CTV‐labeled Tregs (TGF‐β CAR‐transduced or untransduced), and either 0, 5, or 10 ng/mL TGF‐β. The co‐cultures were set up at 1:2:1 Teff:TM‐LCL:Treg ratio. (b) Histogram overlays of CTV dilution comparing co‐cultures containing TGF‐β CAR‐Tregs at varying concentrations of TGF‐β. (c) Histogram overlays of CTV dilution comparing co‐cultures containing untransduced Tregs at varying concentrations of TGF‐β. (d) Histogram overlays of CFSE dilution comparing co‐cultures without Tregs and co‐cultures with TGF‐β CAR‐transduced Tregs, at varying concentrations of TGF‐β

To determine whether CAR‐mediated (as opposed to TCR‐mediated) Treg activation would similarly suppress CAR‐Teff proliferation, a CD19 CAR was introduced into CD4^+^ Teff cells, and co‐cultures were set up with CFSE‐labeled CD19 CAR‐Teff cells and irradiated parental (CD19^+^/OKT3^−^) TM‐LCL target cells, with or without CTV‐labeled TGF‐β CAR‐transduced Treg cells (Figure 7a). In this system, the Teff and Treg cells were separately activated via their CARs by CD19 and TGF‐β, respectively, thus enabling specific inquiry into the effect of CAR activation on the Treg cells’ suppressive potential. CTV dilution in Tregs (Figure 7b) and CFSE dilution in Teffs (Figure 7c) were quantified after 72 hr of co‐culture. Flow cytometry analysis revealed that TGF‐β CAR‐transduced Treg cells, but not untransduced Treg cells, divided in response to TGF‐β addition (Figure 7b,c), thus confirming TGF‐β CAR signaling. However, this Treg population was unable to suppress Teff proliferation (Figure 7d), indicating that CAR‐activated Tregs do not exert significant suppressive influence on nearby Teff cells.

Taken together, these results indicate that even if the starting population for therapeutic T‐cell manufacturing contains Treg cells, transduction with TGF‐β CAR would not lead to the preferential expansion of Treg cells, and the presence of any TGF‐β CAR‐Treg cells is unlikely to induce suppressive effects on the activity of tumor‐targeting Teff cells.

## 4 DISCUSSION

In light of the recently reported clinical utility of shielding adoptive T‐cell therapies from TGF‐β,^13^ further exploration of TGF‐β countermeasures are warranted. We previously demonstrated that TGF‐β–binding CARs can be engineered to rewire Teff cells to proliferate and produce immunostimulatory cytokines in response to soluble TGF‐β, effectively inverting an immunosuppressive signaling molecule to a potent T‐cell stimulant that triggers immunosupportive functions.^14^ Here, we examined how this signal inversion impacts other immune cells that may be found in the tumor microenvironment. We showed that TGF‐β CAR‐T cells can support anti‐tumor immune functions by preserving the cytotoxicity of tumor‐targeting T cells and thwarting the differentiation of bystander T cells into the regulatory phenotype. Furthermore, our results demonstrated that the production of TGF‐β CAR‐T cell products for cancer therapy will not be impaired by the presence of contaminating Treg cells. Specifically, we found that the short (and more potent) TGF‐β CAR does not express well in Treg cells and that Treg cells expressing the long TGF‐β CAR do not exert immunosuppressive effects on Teff cells.

Our experiments showed that at least two mechanisms may contribute to the ability of CD4^+^ TGF‐β CAR‐T cells to counter the immunosuppressive effect of TGF‐β on CD8^+^ cytotoxic T cells. First, experiments with the TGF‐β DNR in the CD20 CAR/Raji system showed that increasing the capacity of CD4^+^ T cells to bind and sequester TGF‐β can reduce TGF‐β–induced suppression of CD8^+^ cytotoxic T cells (Figures 1c,e). Consistent with past studies using soluble TGF‐β–binding proteins, we observed that simple sequestration of TGF‐β can, in some contexts, significantly reduce its immunosuppressive potential.^10^, ^33^ In addition to limiting TGF‐β signaling in immune cells, TGF‐β sequestration may also reduce TGF‐β's ability to promote tumor metastasis via mechanisms such as induction of the epithelial‐mesenchymal transition.^34^ In comparison to the TGF‐β DNR, the TGF‐β CAR provided superior support of anti‐tumor functions in both the CD20 CAR/Raji and NY‐ESO‐1 TCR/M407 experimental systems (Figure 1c–f). This additional anti‐tumor function may arise from the production of immunostimulatory cytokines by activated TGF‐β CAR‐T cells,^14^ which could support not only the engineered CAR‐T cells but also endogenous tumor‐specific cytotoxic lymphocytes. Immunostimulatory cytokines produced by TGF‐β CAR‐T cells may also directly contribute to reducing tumor cell numbers, as TNF‐α and IFN‐γ have been associated with restricted tumor cell growth.^35^ Importantly, in the *in vivo* tumor microenvironment, which includes other cell types absent from our experiments, immunostimulatory cytokines may recruit additional anti‐tumor mechanisms such as the polarization of macrophages and stromal cells toward proinflammatory phenotypes.^36^, ^37^ Therefore, the anti‐tumor contribution of TGF‐β CAR‐T cells may be further enhanced in the more complex *in vivo* milieu. *In vitro* experiments that incorporate additional cell types or *in vivo* experiments with immunocompetent animals would be necessary to fully explore the impact of TGF‐β CAR‐T cells on the tumor microenvironment.

In this study, we also explored whether the presence of Tregs may affect TGF‐β CAR‐T cell production. Most published manufacturing protocols for anti‐tumor T cells have not found it necessary to specifically deplete Tregs.^28^, ^29^, ^30^, ^31^ However, it was unclear whether TGF‐β CAR‐T cell production might be uniquely challenging. Since the CAR responds to TGF‐β, a cytokine known to be produced by activated Treg cells, this posed the theoretical possibility that TGF‐β–secreting Tregs that also express the TGF‐β CAR could enter a positive‐feedback loop driven by self‐activation. Furthermore, while Tregs are typically ignored as a negligible (1%–2%) proportion of PBMCs in healthy donors,^38^ the frequency of Tregs can vary greatly across individuals. High initial Treg frequencies may be of particular concern in cancer patients, where tumor cells have the potential to induce Treg differentiation.^39^, ^40^ Our results show that while TGF‐β CAR‐Tregs can respond to TGF‐β, they do not hinder the proliferation of Teff cells and do not interfere with the stimulation of anti‐tumor T cells.

Regulatory T cells have been engineered to express a variety of CARs targeting immobilized antigens for the purpose of combating autoimmune pathological processes, and CAR signaling triggered by surface‐bound antigens has been shown to induce Tregs to proliferate, secrete immunosuppressive cytokines (including TGF‐β), and repress immune cell activities.^41^, ^42^, ^43^, ^44^, ^45^, ^46^ Results from our present study showed that soluble antigen can also be used to help trigger CAR‐Treg cell proliferation (Figure 7b). However, TGF‐β CAR‐Treg cells do not suppress neighboring Teff cells in response to TGF‐β (Figure 7c). It is possible that TGF‐β CAR‐Treg cells are ineffective at suppressing Teffs because they sequester the immunosuppressive TGF‐β that they produce. However, this hypothesis is blunted by the observation that TCR‐stimulated TGF‐β CAR‐Treg cells were able to inhibit neighboring Teff cells (Figure 6a), indicating that TGF‐β CAR expression alone does not eliminate Tregs’ suppressive potential. Furthermore, TGF‐β production is only one of several mechanisms by which Treg cells can mediate immunosuppression.^47^ An alternative possibility is that the absence of contact‐mediated Treg activation limits the recruitment of contact‐dependent immunosuppression mechanisms that are important in Treg‐mediated immunosuppression.^48^ While the exact mechanism that underlies the lack of suppressive activity in TGF‐β CAR‐activated Tregs remains to be elucidated, our results clearly demonstrate that inadvertent generation of TGF‐β CAR‐Treg cells in a TGF‐β CAR‐T cell product is unlikely to introduce counterproductive immunosuppression.

## 5 CONCLUSION

This study finds that TGF‐β CAR‐T cells can aid anti‐tumor immune functions by shielding neighboring immune cells from the immunosuppressive effects of TGF‐β. We further demonstrate that potential clinical translation of the TGF‐β CAR will not require additional efforts to minimize the Treg compartment, as TGF‐β CAR‐expressing Tregs do not exhibit TGF‐β–induced immunosuppression. Our results support the use of the TGF‐β CAR to rewire T‐cell responses to TGF‐β and reinforce cytotoxic immune functions in adoptive T‐cell therapy.

## CONFLICT OF INTERESTS

Y.Y.C. and Z.L.C. declare competing financial interests in the form of a patent application whose value may be affected by the publication of this work.

## Supporting information

Additional Supporting Information may be found online in the supporting information tab for this article.

Supporting Figure
