# Supplementary figures and images for: TGF‐β–responsive CAR‐T cells promote anti‐tumor immune function
Source: Bioeng Transl Med. 2018 Jul 27;3(2):75–86. doi: 10.1002/btm2.10097 (PMC6063867; doi:10.1002/btm2.10097)

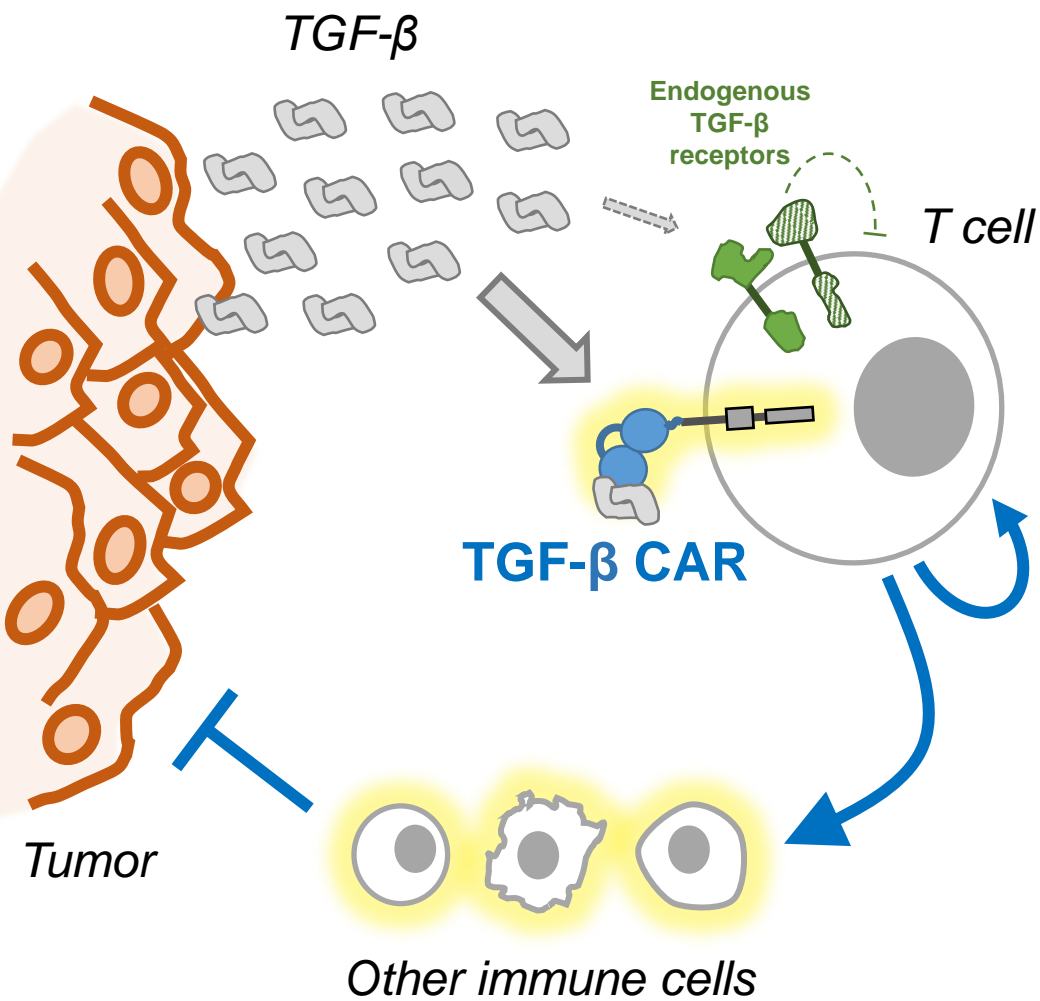

Supplement: Supplementary file 1 — Supporting Figure [file BTM2-3-75-s001.pdf]
